# Hybridization Potential of Two Invasive Asian Longhorn Beetles

**DOI:** 10.3390/insects12121139

**Published:** 2021-12-20

**Authors:** Xingeng Wang, Melody A. Keena

**Affiliations:** 1 United States Department of Agriculture, Agricultural Research Service, Beneficial Insects Introduction Research Unit, Newark, DE 19713, USA; 2 United States Department of Agriculture, Forest Service, Northern Research Station, Hamden, CT 06514, USA; melody.keena@usda.gov

**Keywords:** *Anoplophora*, Cerambycidae, interbreeding, invasive forest pest, subspecies

## Abstract

**Simple Summary:**

Hybridization may occur within individuals of closely related species or species complexes that exhibit similar reproductive systems and behaviors and share overlapping distributions. Successful hybridization results in introgression of genes from one species to another and could significantly modify some essential traits of the hybrids. It is, therefore, important to consider hybridization potential especially among exotic invasive species, which may compromise the implementation of management programs. Asian longhorned beetle (ALB) and citrus longhorned beetle (CLB) are high-risk invasive pests worldwide, attacking various healthy hardwood trees. These two species share some similar host plants and overlapping distributions in large parts of their native ranges in China and the Korean peninsula as well as similar reproductive behaviors. Another longhorned beetle species occurs only in Japan but is considered as a synonym of CLB (JCLB). We found a Chinese CLB population did not cross successfully with a Chinese ALB population, but a JCLB population (male) crossed successfully with a Chinese ALB population (female) to produce viable eggs. We also found CLB crossed successfully with JCLB to produce fertile offspring. This raises potential concern that invasion of these currently isolated species or subspecies into the same regions may facilitate potential inter- or intra-specific hybridization.

**Abstract:**

The Asian longhorned beetle (ALB), *Anoplophora glabripennis* (Motschulsky) and citrus longhorned beetle (CLB), *Anoplophora chinensis* (Förster) (both Coleoptera: Cerambycidae: Lamiinae), are high-risk invasive pests that attack various healthy hardwood trees. These two species share some similar host plants and overlapping distributions in large parts of their native ranges in China and the Korean peninsula as well as similar reproductive behaviors. The original *Anoplophora malasiaca* (Thomson) occurs in Japan and has been synonymized as CLB (hereafter referred to JCLB). In this study, a 30-min behavioral observation of paired adults, followed by a four-week exposure to host bolts, showed that ALB could not successfully cross with CLB. Mating was observed between female CLB and male ALB but not between female ALB and male CLB, no laid eggs hatched. JCLB males successfully crossed with ALB females to produce viable eggs although the overall percentage of hatched eggs was lower than those from conspecific mating pairs. However, ALB males could not successfully cross with JCLB females. CLB and JCLB mated and produced viable hybrid offspring and the hybrid F1 offspring eggs were fertile. These results suggest an asymmetrical hybridization between ALB and JCLB, and that both CLB and JCLB might be considered as two subspecies with different hybridization potential with congeneric ALB. Given their potential impacts on ecosystems and many economically important tree hosts, invasion of these geographically isolated species (ALB and JCLB) or distant subspecies (CLB and JCLB) into the same region may facilitate potential hybridization, which could be a potential concern for the management of these two globally important invasive forest pests. Further studies are needed to determine if fertile hybrid offspring are capable of breeding continually or backcrossing with parental offspring successfully.

## 1. Introduction

Hybridization may occur within individuals of phylogenetically close species or species complexes that exhibit similar reproductive systems and behaviors (e.g., genitalia, mating periods, mate recognition, copulation behavior and sperm use) and share overlapping distributions [1,2,3]. Successful hybridization results in introgression of genes from one species to another and could significantly modify some essential traits of the hybrids (e.g., host range, pheromone composition, thermal requirements, pesticide susceptibility in insects) and even give rise to a hybrid speciation [1,4,5,6,7]. It is, therefore, important to consider hybridization potential especially among exotic invasive species, which may compromise the implementation of management programs. For example, hybridization of *Helicoverpa zea* (Boddie) and *H. armigera* (Hübner) (Lepidoptera: Noctuidae) has resulted in reported decreases in susceptibility to pesticides in *H. zea* and to the proteins of *Bacillus thuringiensis* Berliner (Bacillales: Bacillaceae) in *H. armigera*, as well as increases in the host range for both species [8]. Another notable example is the hybridization of the European honeybee *Apis mellifera* L. (Hymenoptera: Apidae) subspecies with the African honeybee *A. mellifera scutellate* Lepeletier subspecies; both were introduced into the Americas where their hybrids are currently predominant [9].

The Asian longhorned beetle (ALB) *Anoplophora glabripennis* Motschulsky and the citrus longhorned beetle *A. chinensis* Förster (Coleoptera: Cerambycidae: Lamiinae), are among the highest risk invasive forest pests worldwide [10]. Native to China and the Korean Peninsula [11,12], invasive ALB populations were reported for the first time in the US in 1996, in Europe in 2001, and in Canada in 2003, and are still present across Europe and in Massachusetts, New York, Ohio, and South Carolina in the US despite extensive eradication efforts [13,14,15,16]. Genetic analyses suggest multiple introduction events of ALB from China and continuous secondary spread within the invaded area in Europe or the US [17,18,19,20]. *Anoplophora chinensis* is distributed widely in China and the Korean Peninsula and is sympatric with ALB in large parts of these two countries [10,21,22]. It is occasionally found in Indonesia, Malaysia, the Philippines, and Vietnam [10,11], and is also considered present in Japan after the original species *Anoplophora malasiaca* (Thomson) was synonymized with *A. chinensis*, based largely on shared morphological traits of the reproductive system [11]. *Anoplophora chinensis* has been detected in more than 11 European countries since it was first detected in 2000 in Italy [12,23]. Currently, invasive *A. chinensis* populations are still present in Croatia, France, Italy, and Turkey [14,16,23]. Both *A. chinensis* and *A. malasiaca* seem to have invaded northern Italy [24]. *Anoplophora chinensis* was once detected in Washington, US in 2001 but has been eradicated [25]. Both ALB and *A. chinensis* (or *A. malasiaca*) are polyphagous xylophages that attack a wide range of hardwood trees such as *Acer*, *Betula*, *Populus* and *Salix*, and *A. chinensis* (or *A. malasiaca*) has an even wider host range including *Citrus* and some coniferous trees [10,26,27]. They are morphologically similar; the major distinction is the presence of some small tubercles on the basal quarter of each elytron in adult of *A. chinensis* or *A. malasiaca*, but not in adult ALB [10].

Most importantly, ALB and *A. chinensis* (or *A. malasiaca*) share similar reproductive behaviors and life cycles [28,29,30]. Adult females of both species emerge in late spring or early summer (depending on the climate), and newly emerged adults first search for suitable hosts for sexual maturation feeding, then mate, and oviposit in the tree trunk or branches (ALB) or in trunk and exposed roots of tress (*A. chinensis* and *A. malasiaca*) [29,30]. Mate-finding in ALB occurs as the male engages in a sequence of directed searching for the female [31]: (1) both sexes are attracted by host plants at long range via visual cues and host volatiles [1,2,3,4,31,32,33]; (2) upon landing on a tree, males are attracted by female-produced trail pheromones and volatiles from damaged twigs [34,35,36]; (3) males recognize females by visual cues and the female contact pheromone, and quickly mount and attempt to mate [37]. ALB males also produce a pheromone that primarily attracts virgin females and attraction is enhanced by plant kairomones [32,38,39]. When males get closer to females the females may move toward them and even contact them, making it easier for the male to find them. After copulation a pair-bond can last hours [29]. Copulation can significantly reduce the mating receptivity of females, which simultaneously occurs with a reduction of pheromone production [29,34,40].

Like ALB, the *A. malasiaca* females produce contact sex pheromones [41,42] and *A. chinensis* males emit similar male pheromone components as ALB males [43]. Some of those volatile pheromone components in both species are sesquiterpenes originally obtained from the host plants and excreted by the beetles that likely play multiple roles in host location, aggregation, or mate location [32,34,36,38,41,42]. Since adult feeding is a prerequisite to pheromone production and ALB and *A. chinensis* (or *A. malasiaca*) share some same host plants [31,33], host plants could directly contribute to the amount or ratio of pheromone components [31,40]. Multiple studies have also demonstrated an overlap of chemosensory receptors between ALB and *A. chinensis* and a considerable semi-chemical conservation between these species [44,45,46,47].

Because of the phylogenetic relatedness and similar mate-finding cues and reproductive behaviors, these two species may potentially interbreed although this has not been tested previously. It is also unclear if *A. chinensis* and *A. malasiaca* that were originally considered separate species can interbreed. This study aimed to determine the likelihood of hybridization between the two species (ALB and *A. chinensis* (or *A. malasiaca*) and subspecies (*A. chinensis* and *A. malasiaca*) under laboratory conditions. In particular, invasion of these species or subspecies could facilitate hybridization of the populations currently separated geographically in their native ranges such as ALB and *A. malasiaca* and this could have potential consequences for the management of these globally important invasive forest pests.

## 2. Materials and Methods

### 2.1. Insects

All bioassays were conducted in the quarantine facilities of the United States Department of Agriculture (USDA), Forest Service, Northern Research Station quarantine laboratory (NRSQL) in Ansonia, CT or the USDA, Agricultural Research Service (ARS), Beneficial Insects Introduction Research Unit (BIIRU) in Newark, DE. At NRSQL, laboratory colonies of *A. glabripennis* (ALB), *A. chinensis* (CLB) and *A. malasiaca* (JCLB) were maintained under controlled quarantine conditions (25 °C, 60% RH and 16:8 L:D). The ALB colony was established from beetles collected in 1999 in Chicago, IL. The sources of the two *A. chinensis* populations were initially collected in Yancun, Guangdong Province, China and Lombardy, Italy, and maintained at the USDA-ARS, European Biological Control Laboratory (EBCL) in Montferrier-sur-Lez, France. The two CLB populations were imported in 2018 under permit from EBCL. The Italian individuals used were from the 16th, 17th, or 18th laboratory generations and the Chinese individuals were from the 6th or 7th laboratory generations [48]. Molecular analysis of the invasive Italian population indicates its origin from Japan and that it is part of what was previously called *A. malasiaca* prior to the revision of this genus [11,22]. At BIIRU, laboratory colonies of ALB and JCLB were maintained under controlled quarantine conditions (23 ± 1.5 °C, 45–60% RH and 16:8 L:D). The ALB colony was established from beetles collected in Massachusetts, New York, New Jersey and Illinois, USA, and China in 1999 [49]. The JCLB colony was also established from individuals provided in 2018 by EBCL [50].

Rearing methods for these beetles were similar as described for ALB, CLB or JCLB by Keena et al. [48,51] at NRSQL or for ALB and JCLB by Wang et al. [49,50] at BIIRU. Briefly, freshly collected maple tree (*Acer* spp.) branches were cut into bolts (2–5 cm diameter, 15–20 cm long) and used as an oviposition medium for the beetles, while small twigs were used as a food source. Young adult beetles were fed with twigs for about 10 days and then paired for mating. Each pair of adult beetles was reared in a glass jar (3.47 L) by providing 8–10 small twigs as food and one bolt as oviposition substrate weekly. Exposed bolts were held until eggs had hatched (≈one month). Young larvae were excised from the bolts and transferred to 35 × 10 mm petri dishes (Corning Inc., Corning, NY, USA, Falcon ^®^ product #351008) at NRSQL or 28.3 mL plastic cups (SOLO Cup Co., Urbana, IL, USA) at BIIRU for rearing individually on a cellulose-based artificial diet [50,51,52].

### 2.2. Crossing Bioassays at BIIRU

At BIIRU, crossing bioassays were conducted between ALB and JCLB under the quarantine conditions as described above. The experiment consisted of four paired mating treatments: (1) ALB♀ × ALB♂, (2) JCLB♀ × JCLB♂, (3) ALB♀ × JCLB♂, and (4) JCLB♀ x ALB♂. The first two treatments served as controls of conspecific mating while the last two treatments tested potential interspecific hybridization between these two species. Newly emerged and naïve (i.e., no contact with any other adult since eclosion) adults were first individually fed with maple (*Acer* spp.) twigs for about 10 days in a glass jar (3.47 L) and then paired in the jar. Immediately following the pairing, the beetles in these two interspecific crossing treatments (3 and 4) were continually observed for 30 min to record mating behaviors (mounting and copulation). If the male got a full extension of his aedeagus and the copulation lasted close to 2 min, it was considered a successful mating [29]. Each pair was provided with one new maple bolt weekly in their jar for four consecutive weeks and exposed bolts were removed and dissected approximately one month later (i.e., after most viable eggs should have hatched and larvae will be in 1st instar). The number of unhatched (presumed infertile) and hatched (i.e., fertile) eggs were recorded. A sub-sample of 2–5 live larvae from each pair were transferred to artificial diet to rear them to adults as described above. Following the regular rearing procedures, all larvae were reared for 170 days and then subjected to a 120-day cold chilling at 5–10 °C. After the cold chilling the larvae were moved back to the quarantine conditions until pupation and adult emergence. There were 20 replicates for the first two treatments and 25 replicates for the last two treatments. In total, 38 hybrid larvae, 50 ALB larvae and 20 CLB larvae were reared. To compare the fitness of the hybrid offspring with the offspring from the hybrid’s respective parents, we also measured the body weights of mature larvae immediately prior to cold chilling, newly formed pupae, and newly emerged adults as well as the developmental time from post-chilling to pupation and from pupa to adult emergence.

### 2.3. Crossing Bioassays at NRSQL

At NRSQL, crossing bioassays were conducted between ALB and CLB, and between CLB and JCLB under the conditions described above. The experiment consisted of seven treatments of within or between species or subspecies crossing: (1) ALB♀ × ALB♂, (2) CLB♀ × CLB♂, (3) JCLB♀ × JCLB♂, (4) ALB♀ × CLB♂, (5) CLB♀ × ALB♂, (6) CLB♀ × JCLB♂, and (7) JCLB♀ × CLB♂. Direct behavioral observations were conducted for the two between-species (4 and 5) or two between-subspecies (6 and 7) crosses. The mating behaviors of the ALB and CLB have already been published [29,30] and were used to determine what behaviors to record and as the controls for conspecific mating. Each pair was observed for 30 min when they were first paired. A female was first released onto a 1-m-long dry maple bolt, followed by releasing a male just below the female to ensure both the female and male were in proximity. A series of 15 behaviors including approaching, ignoring, following, or avoiding the opposite sex, male antennal wagging, mounting or copulating, were recorded for both sexes. Following the observation, each pair was held together in a jar (3.47 L), provided both food (maple twigs) and an oviposition bolt of red (for CLB or JCLB) or Norway maple (for ALB). The bolts were changed weekly for four consecutive weeks. All eggs were extracted from the exposed bolts and held for hatch. A sub-sample of up to 15 larvae from each successful mating were reared on the diet. Ten pairs were tested for each treatment except for CLB♀ × JCLB♂ and JCLB♀ × CLB♂, in which only 5 and 6 pairs were available for the tests. Adults of the CLB populations were in short supply which limited the number of replicates that was possible. The ages of tested adults ranged from 11 to 64 days old, naïve for all populations except for some ALB males.

Individuals that successfully completed development from the CLB♀ × JCLB♂ (14 females and 16 males) and JCLB♀ × CLB♂ (3 females and 1 male) crosses were mated and hatch was checked for four consecutive weeks to determine if eggs were fertile. There were 13 pairs of mating tests of CLB♀ × JCLB♂ hybrid female and male, 2 pairs of JCLB♀ × CLB♂ hybrid female and CLB♀ × JCLB ♂ hybrid male, and 1 pair of JCLB♀ × CLB♂ hybrid female and male.

### 2.4. Data Analyses

The mortality at early larval stages between the hybrid and parental offspring was compared by the *χ*^2^ Goodness of Fit test. The numbers of eggs laid, or the percentages of eggs hatched were compared among different treatments using a one-way Analysis of Variance (ANOVA). Because these bioassays were conducted under slightly different conditions between BIIRU and NRSQL, the data were analyzed separately. Data for both sexes on the fitness (survival, developmental time, and body size) of the hybrid and parental offspring were pooled because of large variation among individuals. Prior to all ANOVA analyses, proportion data were arcsine square root transformed to normalize the variations after checking for the normality using the Shapiro-Wilk test. For those not meeting the normality assumptions, they were tested using Kruskal-Wallis ANOVA. Multiple comparisons were subsequently performed using Tukey’s honestly significant difference (HSD) test. All analyses were performed using JMP Pro 16 (SAS 2021, Cary, NC, USA).

## 3. Results

### 3.1. Crossing Bioassays at BIIRU

**Figure 1:**
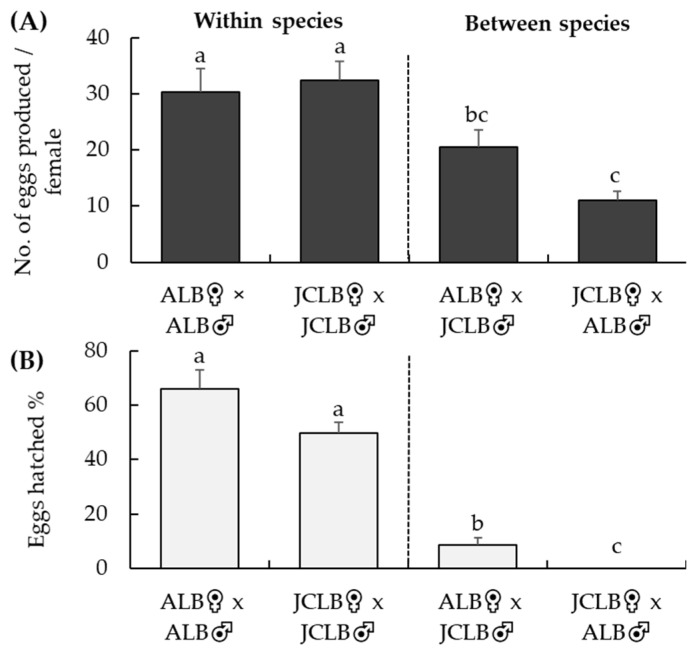
Crossing tests at BIIRU: numbers of eggs laid per female (**A**) *Anoplophora glabripennis* (ALB) and *A. malasiaca* (JCLB) over a 4-week period when paired with a conspecific or interspecific male and percentage of eggs hatched (**B**) in various crossing treatments. Bars refer to mean + SE and different letters above the bars indicate significant difference (ANOVA, *p* < 0.05).

When a female ALB was paired with a male JCLB, mating was observed in 4 out of 25 replicates during a 30-min observation. Mating behavior was not observed when a female JCLB was paired with a male ALB. The total number of eggs laid per female adult during a 30-day exposure to maple bolts was significantly different among the four different crossing treatments (*F*_3,86_ = 10.4, *p* < 0.001) (Figure 1A). Numbers of eggs laid by females were similar between the ALB♀ × ALB♂ and JCLB♀ × JCLB♂ treatments, but a female JCLB laid less eggs when it was paired to an ALB male than to a JCLB male (Figure 1A). Not a single egg hatched from the JCLB♀ × ALB♂ treatment (Figure 1B). About 8% of the eggs successfully hatched from the ALB♀ × JCLB♂ treatment, although this percentage was significantly lower than those hatching from the ALB♀ × ALB♂ or JCLB♀ × JCLB♂ treatments (*F*_3,82_ = 65.1, *p* < 0.001) (Figure 1B).

**Figure 2:**
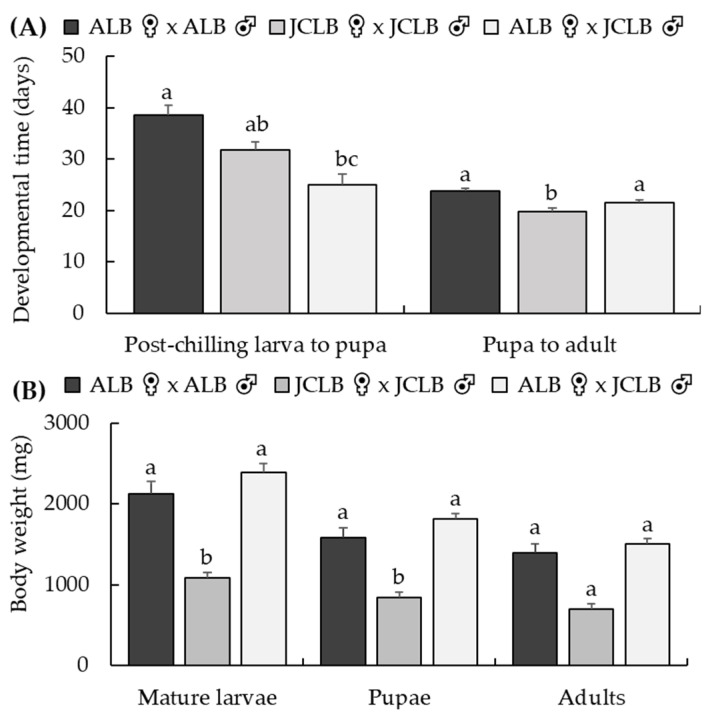
Crossing bioassays at BIIRU: developmental times (**A**) of post-chilled larvae and pupae of *Anoplophora glabripennis* (ALB) and *A. malasiaca* (JCLB) and body weights (**B**) of pre-chilling mature larvae, pupae and adults (B). Bars refer to mean + SE and different letters above the bars indicate significant difference (ANOVA, *p* < 0.05).

**Figure 3:**
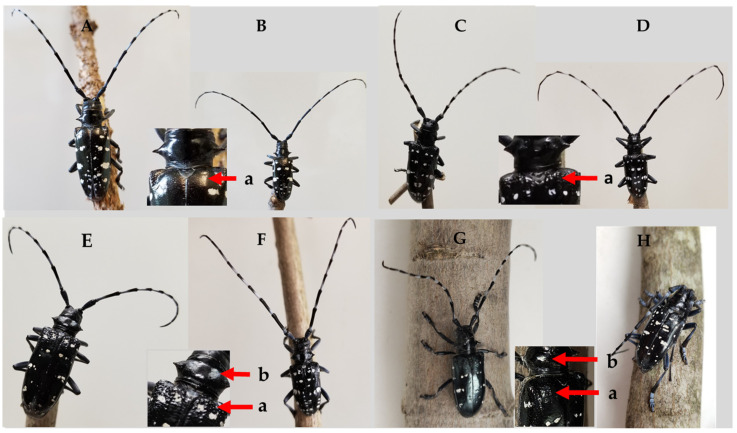
Adult female (**A**) and male (**B**) *Anoplophora glabripennis* (ALB), female (**C**) and male (**D**) *A. chinensis* (CLB), female (**E**) and male (**F**) *A. malasiaca* (JCLB), and female (**G**) and male (**H**) hybrids of ALB and JCLB. Arrows point to the smooth (ALB) or grainy (CLB, JCLB or the hybrid) surface of the basal portion of the elytra (a) or the presence of two white spots on the pronotum in JCLB or the hybrid (b).

Eighteen of the 38 hybrid larvae died at 1^st^ instar while 13 of the 50 reared ALB larvae and 5 of the 20 reared JCLB larvae died at 1^st^ instar. The hybrid larvae mortality (47.4%) was higher than that of ALB (26.0%) (*χ*^2^ = 9.0, df = 1, *p* = 0.003) or JCLB larvae (25.0%) (*χ*^2^ = 10.1, df = 1, *p* = 0.002). Mortality stabilized once the larvae developed into 2nd instar; 17 of the 20 hybrids, 34 of the 37 ALB and 14 of the 15 JCLB larvae eventually developed into adults. The pupation time after cold chilling by the hybrids was shorter than ALB but was similar to that of JCLB (*F*_2,40_ = 13.9, *p* < 0.001) (Figure 2A). There was no difference in the pupal development time between ALB and the hybrids, but JCLB pupated faster than the hybrids or ALB (*F*_2,34_ = 11.4, *p* < 0.004) (Figure 2A). The body sizes of mature larvae (*F*_2,50_ = 28.6, *p* < 0.001), pupae (*F*_2,41_ = 28.1, *p* < 0.001) or adults (*F*_2,34_ = 22.3, *p* < 0.001) of the hybrids were similar to ALB but were smaller than JCLB (Figure 2B). Both hybrid adult male and female resemble JCLB morphologically, with a grainy surface due to small tubercles of the basal portion of the elytra, but unlike ALB whose basal portion of the elytra has a smooth surface (Figure 3). The hybrids, also like JCLB, had two white spots on the pronotum (Figure 3).

### 3.2. Crossing Bioassays at NRSQL

Some typical mate-finding and mating behaviors were observed between ALB and CLB or between CLB and JCLB although the overall frequencies of these behaviors were low during a 30-min observation and highly variable among replicates (Table 1). Between these two species, when a female ALB was paired with a male CLB, although both male and female initiated some contacts, the male largely ignored the female (did not follow or ran away from the female) and no mating was observed. The male more frequently ignored the female in this than the other three crossing treatments (Table 1). When a female CLB was paired with a male ALB, the male approached the female and immediately mounted and initiated a pair-bond if the female was receptive. Between the two CLB populations, mounting and mating were observed more frequently than between the two different species (Table 1). In the crosses between the CLB populations the male followed the female’s trail, or the female followed the male after contacts, and the male wagged antennae and mounted the female.

**Figure 4:**
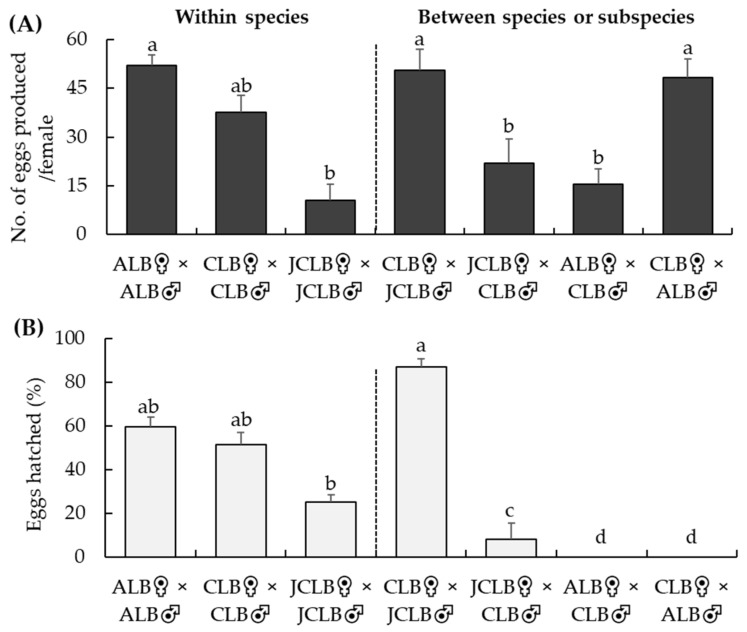
Crossing tests at NRSQL: numbers of eggs laid per female (**A**) *Anoplophora glabripennis* (ALB), *A. chinensis* (CLB) or *A. malasiaca* (JCLB) over a 4-week when paired with a conspecific or interspecific male and percentage of eggs hatched (**B**) in various crossing treatments. Bars refer to mean + SE and different letters above the bars indicate significant difference (ANOVA, *p* < 0.05).

Adult female ALB, CLB and JCLB laid eggs in all these conspecific or interspecific crossing treatments (Figure 4A). However, the numbers of eggs laid varied among the different crossing tests (*F*_6,53_ = 10.6, *p* < 0.001) (Figure 4A). More eggs were produced by females in ALB♀ × ALB♂, CLB♀ × CLB♂, CLB♀ × ALB♂ or CLB♀ × JCLB♂ than in JCLB♀ × JCLB♂, JCLB♀ × CLB♂ or ALB♀ × CLB♂ (Figure 4A). Percentages of hatched eggs were similar among ALB♀ × ALB♂, CLB♀ × CLB♂ and CLB♀ × JCLB♂ but were lower in JCLB♀ × JCLB♂ or CLB♀ × JCLB♂ (*F*_6,48_ = 44.5, *p* < 0.001) (Figure 4B). Not a single egg hatched in ALB♀ × CLB♂ or CLB♀ × ALB♂ (Figure 4B). Mortality of offspring resulting from conspecific mating was 14.0, 9.0 and 8.9% for JCLB (*n* = 340), CLB (*n* = 343) and ALB (*n* = 395), respectively.

The hybrid offspring successfully developed from the CLB♀ × JCLB♂ and JCLB♀ × CLB♂ crosses were fertile. Over the four-week exposure, F1 females of the CLB♀ × JCLB♂ hybrids produced 29.8 ± 6.8 eggs (*n* = 13); 41.1 ± 11.1% of the eggs successfully hatched. One of the two tested F1 females from the JCLB♀ × CLB♂ hybrid female and CLB♀ × JCLB ♂ hybrid male cross produced 34 eggs (79.1% eggs hatched) and one F1 female of the JCLB♀ × CLB♂ hybrids produced 2 eggs (both eggs hatched).

## 4. Discussion

We demonstrated hybridization potential between these two invasive longhorned beetles of Asian origin (ALB and *A. chinensis* or *A. malasiaca*) under the laboratory conditions. A short period of behavioral observations of paired adults, followed with a four-week exposure of the female to host bolts, showed that ALB female did not mate with *A. chinensis* male. Although mating occurred between *A. chinensis* female and ALB male, the female did not produce any viable eggs. However, female ALB and *A. malasiaca* male mated successfully to produce viable F1 offspring, while *A. malasiaca* female and ALB male did not mate. *Anoplophora chinensis* and *A. malasiaca* could mate (either direction) to produce fertile offspring.

It is not surprising that these two species appeared to recognize each another as potential mates because they share some similar pheromones and mate-finding behaviors as well as mating periods and genitalia [10,28,29,30,31]. However, our results showed that *A. chinensis* and *A. malasiaca* differed in terms of the male’s response towards ALB females possibly due to a difference in female contact pheromone composition or other courtship signals that lead to mate recognition. *Anoplophora chinensis* males ignored ALB females and many of them flew away, possibly indicating that the male did not recognize the female, while *A. malasiaca* males mated with ALB females successfully. Our results showed that ALB males approached *A. chinensis* females, and most males mounted and attempted copulation. In contrast, ALB males did not mate with *A. malasiaca* females. This shows not only an asymmetrical hybridization between ALB and *A. malasiaca*, but also differences in the hybridization potential between *A. chinensis* and *A. malasiaca* with ALB. *Anoplophora malasiaca* males showed interest in *A. chinensis* females and mounted but females often rejected the male as if not recognizing them as a potential mate. *Anoplophora malasiaca* females were extremely interested in *A. chinensis* males who appeared not to recognize them as potential mates and often ignored the females.

There is still controversy over the synonymy of *A. chinensis* and *A. malasiaca* because they can be differentiated based on the presence (*A. malasiaca*) or absence (*A. chinensis*) of two white spots on the pronotum (Figure 3) and by their mitochondrial COI haplotypes [53,54]. Morphological variations can exist among different geographical populations. For example, the elytra of ALB adults are marked with white spots in some populations but yellow in other populations in China and the original yellow type *Anoplophora nobilis* (Ganglbauer) was considered as a synonym ALB (white type) as cross-mating experiments between these two types yielded viable offspring and intermediate colors of the patches of setae on the elytra of the hybrid offspring adult [55]. Recent studies also showed a difference in thermal responses between *A. chinensis* and *A. malasiaca* [48,56]. Despite these differences, our results first confirm that *A. malasiaca* is reproductively compatible with *A. chinensis* (i.e., considered as one biological species). We thus suggest considering them as two subspecies. Genetic analyses suggest that *A. malasiaca* originated from *A. chinensis* in the Asian continent and immigrated into the Japanese Archipelago through the Korean Peninsula at least twice in the past [53]. These differences may arise simply as a result of geographic isolation over time.

The number of eggs produced per female over the four-week exposure varied among different crossing treatments. Tests conducted at BIIRU used same aged and young female beetles and showed no significant difference in the number of eggs laid by female ALB and *A. chinensis*, but females of both species laid more eggs when they were paired with conspecific males than those paired with interspecific males. In the tests conducted at NRSQL, *A. malasiaca* females laid less eggs than ALB or *A. chinensis* females in both conspecific and interspecific crossing tests. We must emphasize that the age of these females used in different tests at NRSQL varied due to the lack of available insects. Female age is known to affect the fecundity of these beetles [28,29,48]. However, our particular interest was to show that female ALB, *A. chinensis* or *A. malasiaca* still laid infertile eggs even in the absence of mating.

We found that crossing between *A. malasiaca* male and ALB female produced some viable eggs although the overall percentages of hatched eggs were low (ranged from 4.3 to 30.3%) when compared to the results of conspecific crossing tests. Even though copulations were observed or could occur during the four-week exposure, not all eggs may be fertilized. The hybrid progeny from ALB and *A. malasiaca* appeared to be no different than those of their respective parental offspring in terms of body size and developmental time, except that the hybrid offspring had relatively higher mortality at the earliest larval stage. However, in nature, if females can remate with conspecific males, sperm from the conspecific males could be more competitive than that of the heterospecific males in multiple-mated females [1,3]. It is also possible that when a female is inseminated with heterospecific sperm first, these females may have reduced or no receptivity towards conspecific males [1]. Therefore, the interspecific mating may have minimum measurable effects in nature if it occurs.

Various factors such as phylogenetic relatedness, geographical distribution, spatial and temporal barriers to mating, mate recognition, copulation and sperm use, hybrid inviability and sterility can affect the success of hybridization [1,3]. Introgression could also alter morphs, genomes, behaviors, endogenous pheromonal components, thermal tolerance, pesticide resistance, host range of hybrid offspring as well their interaction with natural enemies and have important ecological and pest management implications, especially for invasive agricultural and forest insect pests [7,8,9,57,58]. In their native rage, ALB and *A. malasiaca* or *A. chinensis* and *A. malasiaca* remain geographically separated. Both ALB and *A. chinensis* (or *A. malasiaca*) have been intercepted continually in Europe and North America in wood packing materials and/or live plants and therefore new invasions are ongoing concerns [10]. Invasion of both species or subspecies into the same regions could facilitate potential interspecific or intraspecific hybridization. If fertile hybrid offspring are capable of breeding continually or backcrossing with parental offspring successfully, potential interbreeding could result in genetic variations of the parent species population or even give rise to a hybrid speciation [4,5,6,59]. This would also pose significant implications for the systematics of this group and implementation of management programs.

In summary, we evaluated interbreeding potential between these two invasive Asian longhorned beetles. Although larger sample sizes for the behavioral observations and crossing tests would render a more reliable estimation of statistical significance, these data confirmed the occurrence of interbreeding between these two species, and between *A. chinensis* and *A. malasiaca* under laboratory conditions. Currently, studies are ongoing to determine if the hybrid offspring of ALB female x *A. malasiaca* male are fertile through backcrossing them with their respective parental species. Further detailed mating studies and genomic comparisons are also required to determine genetic compilation, incompatibility, morphological and biological characteristics of the hybrids as well as possible consequences on the management strategies (e.g., detection, biological control). For example, is the hybrid offspring suitable for the host specific egg parasitoid *Aprostocetus fukutai* (Hymenoptera: Eulophidae) of *A. chinensis* or *A. malasiaca* that does not develop in ALB eggs [50,60]? Do hybrids have different thermal performance than parental offspring [48,56] and what pheromones are produced by hybrids?

## Figures and Tables

**Table 1:** Frequencies of reproductive behavioral events between Asian longhorned beetle (ALB), *Anoplophora glabripennis* and citrus longhorned beetle (CLB) *A. chinensis* or between CLB and *A. malasiaca* (JCLB) during a 30-minitue observations.

Mating Behaviors	ALB♀ × CLB♂ (10) ^1^	CLB♀ × ALB♂ (10) ^1^	CLB♀ × JCLB♂ (5) ^1^	JCLB♀ × CLB♂ (7) ^1^	*F*_3,27_	*p*
Female approaches male	0.60 ± 0.43 a	0.50 ± 0.31 a	0.40 ± 0.24 a	3.29 ± 1.13 b	5.62	0.004
Male ignores female	0.50 ± 0.40 a	0.20 ± 0.13 a	0.20 ± 0.20 a	2.43 ± 0.75 b	5.76	0.004
Male follows female	0.00 ± 0.00 a	0.10 ± 0.10 a	0.00 ± 0.00 a	0.14 ± 0.14 a	0.64	0.593
Female follows male	0.30 ± 0.30 a	0.20 ± 0.20 a	0.00 ± 0.00 a	1.43 ± 0.61 b	3.68	0.024
Female runs or flies	0.20 ± 0.13 a	0.20 ± 0.13 a	0.00 ± 0.00 a	0.14 ± 0.14 a	0.41	0.750
Male approaches female	1.50 ± 0.27 a	1.60 ± 0.52 a	1.20 ± 0.20 a	1.86 ± 0.40 a	0.33	0.805
Male ignores female	0.80 ± 0.13 b	0.30 ± 0.21 a	0.00 ± 0.00 a	0.29 ± 0.18 a	3.23	0.038
Male follows female	0.00 ± 0.00 a	0.30 ± 0.30 a	0.00 ± 0.00 a	0.29 ± 0.18 a	0.73	0.543
Female follows male	0.10 ± 0.10 a	0.10 ± 0.10 a	0.00 ± 0.00 a	0.14 ± 0.14 a	0.22	0.884
Male runs or flies	0.70 ± 0.33 a	0.10 ± 0.10 a	0.00 ± 0.00 a	0.71 ± 0.18 a	2.27	0.103
Male antennal wagging	0.00 ± 0.00 a	0.30 ± 0.15 a	1.00 ± 0.00 b	0.14 ± 0.14 a	13.14	<0.001
Female receives male	0.00 ± 0.00 a	0.10 ± 0.10 a	0.00 ± 0.00 a	0.00 ± 0.00 a	0.80	0.506
Female rejects male	0.00 ± 0.00 a	0.60 ± 0.22 ab	1.00 ± 0.00 b	0.43 ± 0.20 ab	5.48	0.005
Male mounts female	0.00 ± 0.00 b	0.90 ± 0.31 a	1.00 ± 0.00 b	0.43 ± 0.20 ab	4.36	0.013
Male copulates female	0.00 ± 0.00 a	0.20 ± 0.13 a	0.60 ± 0.40 a	0.43 ± 0.30 a	1.86	0.160

^1^ Values are mean ± SE and different letters within a row indicate significant differences (*p* < 0.05). Numbers in parenthesis are replicates of pairs observed.

## Data Availability

Data are available upon request.
